# Navigating the practice of co-produced convergence research: Lessons from projects funded by NSF’s Coastlines and People (CoPe) program

**DOI:** 10.1017/cft.2026.10048

**Published:** 2026-08-11

**Authors:** Jenna H. Tilt, Mona V. Behl, Laura J. Moore, Samantha L. Mosier, Lisa Auermuller, Rebecca Zarger

**Affiliations:** 1 College of Earth, Ocean, and Atmospheric Sciences, Oregon State University, USA; 2 William and Mary, Virginia, USA; 3 The University of Georgia, Georgia, USA; 4 The University of North Carolina at Chapel Hill, USA; 5 East Carolina University, USA; 6 Rutgers University New Brunswick, USA; 7 University of South Florida, USA

**Keywords:** coastlines, collaboration, convergence, co-production, people, resilience

## Abstract

Convergent, co-produced coastal research engages researchers across disciplines and brings together researchers, practitioners and community representatives to co-create new knowledge and understanding to support community resilience while advancing scientific understanding. This type of research is increasingly required by funding agencies and yet it is relatively new. Given this, along with the expansive range of scholarly and lived expertise required to carry out this type of research, there are many epistemological, relational, temporal and institutional challenges for successful projects to overcome. Here we describe these challenges, along with strategies employed to overcome them, based on the work of multiple convergent, co-production research projects focused on coastal communities and environments, funded by the National Science Foundation’s Coastlines and People (CoPe) Program. Our experience leads us to recommend that project leaders use inclusive communication practices; incorporate boundary spanners; build shared understanding; select the style and depth of co-production intentionally; focus on earning trust; tailor co-production approaches to the communities involved; equalize power dynamics; embrace and acknowledge the expanded timeline required; plan for delays; embrace flexibility; and build and sustain partnerships for the long haul. Additionally, supporting this type of work requires academic institutions to reward and value the best research practices and research products that emerge from convergent, community-engaged research, including revision of promotion and tenure guidelines to accommodate the expanded timelines required, and the different types of outcomes and products arising from these types of efforts. Further, we recommend that funders consider offering staged, progressive funding opportunities and longer funding timeframes, in recognition of the time it takes to build and sustain meaningful, trusting community partnerships and to plan research projects in a deeply collaborative way with community partners.

## Impact Statements

This article reflects on ways to navigate the complexities involved in convergent, co-produced coastal research, a high-stakes type of work in which knowledge production and use depend on – and are integrated with – deep community partnerships grounded in shared values and goals. By identifying specific challenges – such as rigid academic timelines, divergent disciplinary lenses and power imbalances – this overview offers actionable strategies for project leaders, universities and funding agencies that support collaborative, co-produced convergence research.

## Introduction

Coastal zones worldwide face unprecedented pressures from intersecting economic, ecological and infrastructural demands amidst rapidly changing environmental conditions, including accelerating sea level rise, increasing precipitation and rising temperatures, among other challenges. Convergent research approaches are critical to address the growing risks faced in coastal areas around the globe; however, the practicalities and ethics of successfully integrating research and problem-solving across disciplines present many challenges. Additionally, although researchers and funders increasingly recognize the need to co-produce knowledge and understanding through deep community engagement with stakeholders (e.g., government agencies, boundary spanners), holders of indigenous knowledge (e.g., Indigenous populations and Tribes) and communities (e.g., residents, community-based organizations, business), there has traditionally been limited support for such endeavors.

The National Science Foundation’s Coastlines and People Program (CoPe; established in 2019) sought to combine convergent research and co-production of knowledge by requiring the development of projects that integrate contributions from universities and a diverse set of community members and partners to co-develop fundamental, societally relevant research in support of coastal communities. The CoPe Program represents an institutional response to compound challenges experienced by coastal communities, operationalizing what the National Academies of Science, Engineering and Medicine have termed “convergence research” principles (NASEM, 2019; Finn et al., 2022).

Convergence research is defined by NASEM as “an approach to problem solving that cuts across disciplinary boundaries [by] integrating knowledge, tools and ways of thinking from multiple disciplines including natural and social sciences, engineering and others to form a comprehensive framework to address scientific and societal challenges that exist at the interface of multiple fields. By merging these diverse areas of expertise in a network of partnerships, convergence research stimulates innovation from basic science discovery to translational application (National Research Council, 2014, p. 1 paraphrased here).” Convergence research inherently demands deep integration of knowledge, theories, methods, data and perspectives across diverse disciplines to address specific and compelling problems (NASEM, 2019; Peek et al., 2020). This kind of research goes beyond interdisciplinary collaboration (Sharp et al., 2016) to create new ways of thinking and solutions only discoverable by disciplines meeting at the edge of their respective fields (Nash, 2008; Reid et al., 2016; Peek et al., 2020). Yet how to effectively enable and sustain convergence research is a topic of continued inquiry. The National Research Council identified four key challenges to convergence research: (i) establishing effective organizational cultures, structures and governance; (ii) addressing faculty development and promotion needs; (iii) creating education and training programs; and (iv) forming stakeholder partnerships (NASEM, 2019, pp. 60–61). While much progress has been made, these challenges still remain (Petersen et al., 2021; Bammer et al., 2023; Ashton et al., 2024; Berkes et al., 2024).

Like convergence research, co-production of knowledge is a collaborative process that involves a diversity of stakeholders (e.g., government agencies, boundary spanners), holders of indigenous knowledge (e.g., Indigenous populations and Tribes) and communities (e.g., residents, community-based organizations, business) working together on equal footing with researchers to create shared value, often blending resources, expertise or perspectives to achieve a common goal (Kates et al., 2001; Wyborn et al., 2019; Norström et al., 2020; Yua et al., 2022; Muhl et al., 2023). Muhl et al. (2023) describe the process as “bringing a plurality of knowledge sources and types together to formulate and to address a defined problem,” which points to a widely shared “solutions” framing as an outcome of co-produced knowledge. The University Corporation for Atmospheric Research (UCAR) Coastlines and People (CoPe) Synthesis Report underscores the need for co-produced research that incorporates community needs and priorities to identify evidence-based solutions that incorporate and preserve cultural knowledge and ecological processes (University Corporation for Atmospheric Research [UCAR], 2020). The report provides suggestions for developing and sustaining co-production practices such as mixed science advisory groups, broadening access to grants, setting standards for community engagement practices and establishing mentoring practices for community science data collection (University Corporation for Atmospheric Research (UCAR), 2020). However, understanding how to co-produce research knowledge without overburdening practitioners and residents as well as academics, while also creating equitable opportunities for participation remains a challenge (Lemos et al., 2019; Welch-Devine et al., 2025).

The aim of the paper is to collectively consider the strategies used as well as the challenges inherent in the pursuit of convergence research that actively engages with co-production of knowledge and understanding. To accomplish this, we draw from our collective experience as researchers engaged in six different CoPe projects, with varying aims, scales and levels of funding support (see Table 1). Funded projects range from research coordination networks (RCN, ~$500 K projects) to focused hubs (~$5 M projects) to large-scale hubs (~$20 M projects) with a broader reach and scope. Here, we describe the challenges we encountered in co-producing convergence research across all three types of CoPe projects, and suggest strategies and recommendations for researchers, institutional leadership and funding agencies to assist in overcoming these challenges. The examples we provide are derived from our experiences as members of the CoPe projects listed in Table 1, and identified by superscripts throughout. Collectively, the insights we share provide both a constructive assessment of current hurdles or limitations to successful co-production of convergence research, as well as the strategies employed to overcome these hurdles within the context of coupled human-natural coastal systems, yielding a potential framework for realizing the National Academies’ vision of convergence science (NASEM, 2019).

**Table 1.** CoPe projects that are represented in this manuscript Table 1. long description.

Title of the project	Kind of CoPe	Size of CoPe award	Original award timeframe	Geographic scope	Goal(s) of the project	Key disciplines engaged in convergent activities and relevant products	Co-production partners
The Cascadia coastlines and people hazards research hub^1^	Large-scale CoPe hub	~$20 M	5 years	Cascadia (WA, OR and northern CA coastal communities)	The goal is to contribute to socioeconomic viability and equitable resilience in service of coastal community identities, needs and values through targeted scientific advances. Inform and enable integrated hazard assessment, mitigation and adaptation – including comprehensive planning, policy making and engineering – co-produced in sustained collaboration with coastal communities.	Applied Economics, Applied Mathematics, Civil Engineering, Computer Science, Geography, Geology, Geomorphology, Planning, Public health, Public policy, Seismology and Social Science *Key convergent papers:* Dunham et al., 2025 Meselhe et al., 2026 Taherkhani et al., 2026	**Federal:** USGS, NOAA, USACE **Regional:** NANOOS PNSN Environmental consultants Tribal Governments in WA, OR, and Northern CA WA, OR and CA Sea Grant **State:** Emergency management Private Foundations Public Health Geology Natural Resources Transportation **Local (County/City):** Emergency Management Planning Parks and Recreation School Districts **Local:** Community-Based Organizations Churches Homeowner Associations **Academic institutions:** University of Washington Washington State University Oregon State University University of Oregon Cal Poly Humboldt
Megalopolitan coastal transformation hub (MACH): Researching complex interactions between coastal hazards and Risk^2^	Large-Scale CoPe Hub	~$20 M	5 years	New York City, coastal New Jersey and Philadelphia	The goals are: (i) to do science that is useful and used, (ii) to do science that advances understanding of how coastal climate hazards, landforms and human decisions interact, (iii) to train the next generation of leaders in transdisciplinary climate research and engagement and (iv) to learn how to do the first three goals better.	Climate and sea-level science, impacts and adaptation; geomorphology; urban planning and design; emergency management; civil engineering; ethics; economics; public policy; and decision analysis	City of Philadelphia, state of New Jersey – department of environmental protection, local community-based non-profits
Strong coasts: Developing Solutions with nature and people^3^	Large-scale CoPe Hub	~$20 M	5 years	Belize, Florida (Miami), U.S. Virgin Islands	Strong coasts aims to co-create nature-based solutions to coastal hazards on reef-lined coasts with an emphasis on coral and mangrove protection, erosion and flooding. Co-production goals are to collaboratively amplify local ecological knowledge, community experiences of coastal environmental change, and to document community priorities for nature-based solutions in policy arenas. Project outcomes integrate community members directly into coastal policy making and localized risk-response strategies.	Anthropology, Geosciences, Environmental engineering, Marine sciences, Coral restoration, Mangrove restoration, Coastal conservation, Climate adaptation and resilience, Dynamic and static socioecological modeling, Environmental economics, Risk and hazards modeling, Environmental social sciences *Key Convergent Papers:* Hinds et al., 2025; Grace-McCaskey et al., 2026; Reguero et al., 2026	—Project Co-PIs at World Wildlife Fund–Belize and Fragments of Hope (local NGO in Belize) – Community leaders, residents, knowledge holders and local experts – Black in Marine Science (BIMS) – National, regional and local level environmental, coastal and community institutions and organizations in each site Government ministries and institutes (Belize) Federal agencies (Miami and USVI) National, regional and local coastal resilience, restoration and conservation organizations Village councils, Fishers cooperatives, environmental action groups —Primary, secondary and tertiary educational institutions in sites —Cultural heritage and community-based organizations —Seven academic institutions (Univ. of South Florida, UC Santa Cruz, Stanford, Univ. of Miami, Boston Univ., Univ. of the Virgin Islands, East Carolina Univ.)
Supporting environmental Justice in connected coastal communities through a regional approach to collaborative community science^4^	Focused Hub	~$5 M	5 years	Eastern North Carolina	The goal is to understand how social and environmental systems intersect and contribute to risk and resilience in eastern North Carolina communities. This is done through coordination, collaboration and co-production of scientific and programmatic activities among academics, community partners and government actors.	Anthropology; biology; data science; earth system science; (behavioral) decision science; economics; engineering; entrepreneurship; geography; geology; marine science, planning; political science; psychology; public policy and administration; science education; sociology *Key Convergent Paper:* Vance-Chalcraft et al. (2024)	Academic researchers and faculty; community non-profits; community leaders, local and state officials; residents including youth
Building a Collaboratory for coastal adaptation over space and time (C-CoAST)^5^	RCN	~$575 K	2 years	North Carolina, Nationwide, global	The goal of C-CoAST is to enable the potential to steer away from future outcomes that communities wish to avoid and toward outcomes they deem more desirable.	Geoscience, hydrology, biogeochemistry, economics, political science, physics, sociology, urban planning, education *Key Convergent Paper:* Franklin et al., 2026	NC SeaGrant; North Carolina office of resilience and recovery; NC Division of coastal management; U.S. Army Corps; Representatives and government officials from communities along the NC Coast; University of North Carolina; Duke University; University of North Carolina at Wilmington, East Carolina University, NC State University
People on the Move in a Changing Climate (PEMOCC)^6^	RCNs	~$500 K	3 years	Georgia, Virginia, Nationwide	This Research Coordination Network (RCN) leverages relationships with local communities to facilitate collaborations among the broader scientific enterprise. Practitioners, resource managers and coastal stakeholders will coordinate with researchers to understand coastal change impacts on human mobility.	Earth system science, social science, engineering, law and policy *Key Convergent Paper:* Kolesar et al. (2024)	The National Sea Grant College Program, Academic experts engaged in migration research across the nation, practitioners, planners, community leaders, non-profits

1 *Notes*: Cascadia CoPe Hub – https://cascadiacopeshub.org.

2 MACH – https://coastalhub.org/.

3 Strong Coasts – https://www.nsf.gov/awardsearch/show-award/?AWD_ID=2209284.

4 ECU FH – https://www.nsf.gov/awardsearch/show-award?AWD_ID=2052889.

5 C-COAST – https://c-coast.org/.

6 PEMOCC – https://www.nsf.gov/awardsearch/show-award/?AWD_ID=2619130.

The range of project reach and scope represented in our analysis allows us to capture how co-production barriers shift as a function of project complexity. Within smaller scale networks like RCNs, the primary barriers often center around maintaining long-term stakeholder momentum and transitioning from agenda-setting to active implementation given the lack of guarantee of future program or institutional funding. Conversely, the focused and large-scale hubs wrestle with the bureaucratic friction of balancing multi-tiered institutional governance, integrating highly disparate datasets and avoiding community engagement fatigue across broad geographic regions. By examining co-production through this diversified lens, the barriers and strategies we outline are not one-size-fits-all, but are rather those we witnessed and employed while working with different levels of funding, geographic reach and organizational design.

The analysis of the efforts of these six projects presents a unique opportunity to consider the patterns that have emerged from engaging in multi-year research projects that center *both* convergence (across traditional disciplinary silos) and co-production (joint creation of knowledge and practice between stakeholders, holders of indigenous knowledge and communities). We explore previous definitions and approaches to these concepts as well as our working definition drawn from commonalities across our respective projects in more detail below. The CoPe program originated from a series of workshops that grappled with ways to bring disparate fields together to more effectively address seemingly intractable challenges in coastal areas experiencing dramatic socioecological change. As the terms convergence and co-production often appear together in recent high-investment, high-profile transdisciplinary and interdisciplinary programs funded by federal agencies, greater attention to the scoping, common ethical and methodological challenges and opportunities such work affords will enhance future synergistic programs with similar goals.

## Insights: Challenges to co-production of convergence research

The path to equitable academic-community partnerships is fraught with systemic hurdles and challenges that emerge from decades of individual and institutional practices that were not designed with co-produced convergence research in mind and are therefore ill-suited to support it (e.g., Morton et al., 2015; Pischke et al., 2017; Finn et al., 2022). Here, we identify challenges that manifest across four dimensions (epistemological, relational, temporal and institutional), each compounding the others, potentially hindering meaningful collaboration and solutions in coastal research.

### Epistemological challenges

Epistemological challenges emerge when different knowledge systems collide (Petrie, 1976; Miller et al., 2008; Nightingale, 2016; Boon and Van Baalen, 2019). For example, when an ecologist discusses ecosystem services delivered by coastal wetlands and marshes alongside a policy or planning expert analyzing land-use regulatory frameworks, semantic differences often reflect fundamentally distinct approaches to knowledge construction. Without intentional efforts otherwise, these divergences can span vocabulary, methodologies and even basic assumptions about evidentiary standards, impeding clear communication, understanding and meaningful collaboration.

The challenge of convergent research persists even within seemingly unified fields or areas of research. For instance, marine biologists quantifying fishery stocks and marine economists assessing market values frequently employ different terminology for identical phenomena. Such challenges, which are ubiquitous across fields, are a byproduct of disciplinary training that emphasizes specialized communication styles and may have differing value systems. The challenges exponentially increase with collaboration across traditional disciplinary boundaries, such as between natural and social scientists^6^, engineers and environmental scientists. Although convergent work is possible, diversity of expertise on a project becomes problematic when teams conflate shared terminology with shared understanding, often overlooking how different disciplines define concepts like “uncertainty,” “data quality” or “research rigor.” Indeed, Choi and Richards (2017) highlight that the U.S. National Academies (2005) emphasizes communication as key and at the heart of interdisciplinary research and even more so with collaboration between scientists and members of local communities.

Co-produced research also faces profound cultural and linguistic challenges that extend beyond academia, as even the most well-meaning projects can fail due to unexamined assumptions and communication gaps. Similar to interdisciplinary research, collaboration between scientists, stakeholders, holders of indigenous knowledge and community members can also falter amidst miscommunication. For example, local knowledge systems may define “critical” infrastructure differently than regulatory frameworks or researchers in engineering. These perceptual differences can lead to a mismatch between the community infrastructure prioritized for hazard mitigation by regulators and infrastructure most desired by the community (Stanton and Tilt, 2023). Disciplinary silos can also foster incompatible definitions, methodologies and standards of evidence that frequently conflict with communities’ lived experiences and traditional ways of knowing. For example, rationalists and positivists hold differing attitudes and values toward co-production and community engagement compared to a constructivist (Riccucci, 2010). These divides often remain unaddressed because few researchers receive training to navigate such fundamental differences in worldviews (Choi and Richards, 2017) or how to manage epistemological pluralism (Miller et al., 2008; Boon and Van Baalen, 2019).

For example, in work led by one CoPe team^6^ that focuses on climate-induced human mobility, the project team learned that words such as migration, displacement, managed retreat and relocation can mean different things to different communities. The team also learned that ideas proposed in support of one “solution,” such as managed retreat, may not attend to a community’s need to maintain deep cultural ties to ancestral lands, undermining trust and cooperation. This finding is similar to those by Siders et al. (2019) and Agyeman et al. (2009) that highlight the need to focus on the emotional, psychological and symbolic needs of a community and tailor responses and interactions to fit community needs.

For another CoPe team, coming to consensus on what constitutes “participatory” research with “community members” was difficult when differing definitions of “community” were held by researchers from different fields and institutions. This led to differences in team members’ assessments of whether or not participants in particular workshops were “representative enough” of those living in the study site locations to be considered “the community.”

In yet another CoPe project^2^, the meanings of terms such as “co-production,” “stakeholder,” “community” and “risk assessment” were different among project participants, leading to tensions among different disciplinary teams. Such epistemological pluralism and lack of familiarity with other knowledge production processes can inhibit productivity in interdisciplinary and convergent-based research teams (Miller et al., 2008; Choi and Richards, 2017; Boon and Van Baalen, 2019). These challenges represent more than semantics; they can impact the conclusions drawn from and the guidance provided by co-produced research.

In addition to challenges related to terminology and meanings associated with different aspects of co-production, developing new partnerships with culturally diverse community members and community-based organizations that have not participated in academic work means that key aspects of research – such as institutional review board (IRB) processes, participant consent as well as realistic timeframes for research outcomes – may be unfamiliar to community participants. These challenges, further discussed in the next sections, can also occur within cross-disciplinary teams that include researchers who have not previously been involved in human subjects-based research.

### Relational challenges

Convergent, co-produced research requires a diversity of team members who need to work together well, yet developing cohesive working relationships can be challenging for a variety of reasons. One primary challenge is acknowledging and defining co-production of research among science teams. There are many different types of co-production (Meadow et al., 2015, Chambers et al., 2021, Fleming et al., 2023) and different projects or project elements may be better suited to one model than another.

Meadow et al. (2015) categorize co-production research engagement approaches as ranging from more one-way consultative processes such as rapid assessment, where researchers seek to understand how stakeholders frame an issue, to two-way partnerships or what the authors referencing Biggs (1989) call “collegial” approaches that seek to understand complex human-built-environmental implications of some phenomena, such as coastal adaptation strategies. However, other scholars have argued that true co-production of knowledge cannot be one-way but always requires two-way exchanges of ideas and knowledge systems that are on “equal footing” (Yua et al., 2022; Welch-Devine et al., 2025). This higher order of engagement focuses on understanding and developing local capacity for research (e.g., providing the resources, training and tools to effectively and equally co-produce research) to co-generate applied research outcomes (Meadow et al., 2015; Welch-Devine et al., 2025).

Even if researchers understand and appreciate the wide range of modes of co-production, not all researchers on large convergent science teams like those of CoPe may be comfortable with or truly capable of carrying out co-produced research without additional training. Community-engaged, co-produced research requires skills that many scientists lack formal training in, including conflict resolution and consensus-building. While some graduate students and postdocs enter community-engaged projects with relevant training, such preparation remains uncommon in many graduate programs, particularly in the natural sciences. Some disciplines, such as anthropology, geography and sociology, offer time-tested frameworks for effective participatory research, including methods to assess and improve legitimate co-production approaches such as tracking continued participation and representativeness of that participation (e.g., demographics, positionality) and assessing community members’ and partners’ experiences participating in co-produced research (David-Chavez et al. 2024; Welch-Devine et al., 2025). However, many graduate students in those disciplines are unfamiliar with interdisciplinary team-science approaches to research.

Like research teams and researchers, communities also vary greatly from one another and are composed of individuals having diverse backgrounds, perspectives and opinions. Thus, each community has its own unique challenges and opportunities when co-producing research and knowledge with researchers. For example, communities with more capacity (e.g., financial resources, staff, community support) may be more willing or able to participate in co-produced research, while under-resourced communities may lack the capacity to participate. The imbalance between differently resourced communities can perpetuate over time as community–research partnerships strengthen and create a self-reinforced feedback loop, leaving under-resourced communities with potentially fewer opportunities to benefit from co-produced research (Castán Broto et al., 2022; Akerlof et al., 2023). Additionally, there are often priorities and needs in communities – well-resourced or not – more pressing than the research topic at hand. Some argue that the broad goals of co-produced research for coastal adaptation planning, need to be embedded in and address local needs such as affordable housing and workforce development (Helgeson et al., 2024).

Any model of co-production of knowledge requires trust between researchers and project partners. This can be challenging given deep mistrust of researchers rooted, understandably, in examples of historical exploitation through colonialism and extractive research (Gordon and Him, 2025). From well-known historical examples of research that did not involve informed consent to modern-day examples of extractive science, many communities have learned through painful experiences to regard academic collaborations with warranted caution (McElfish et al., 2021; Parsons et al., 2021). These concerns can be unintentionally reinforced; for example, if community members are relegated to roles in which they are less than an equal partner in the research, or if consent processes are rushed, replicating the very trauma researchers are claiming to address. Even well-intentioned participatory projects can stumble at the threshold of trust, especially when institutional credentials or academic titles are assumed by researchers and other research partners to be sufficient for establishing genuine rapport. Potential power imbalances between researchers and project partners can further exacerbate distrust (Akerlof et al., 2023).

### Temporal challenges

A considerable challenge associated with co-producing convergent research is the mismatch between the timeframe from release of requests for proposals (RFPs) to submission and the time needed to develop a meaningful co-produced large-scale research program with stakeholders and communities who can benefit from the work being proposed. This can limit successful grantees to those who have already established the relationships necessary to carry out the work an RFP is intended to inspire or limit the degree to which community partners can be included in a meaningful way in project development. Relatively smaller CoPe awards such as NSF RCN and EAGER (EArly concept Grants for Exploratory Research) grants were intended to address this concern by providing time for teams to build relationships, plan work and carry out pilot projects. Yet, in the end, this intention was not realized because the request for proposals for the larger CoPe awards was issued only six months after the initial smaller grants were awarded. This meant that, in some cases, teams already engaged in co-production and community-driven work had an advantage in preparing successful proposals, and the time available for all applicants to co-produce project plans and proposals with community partners was limited, constraining some aspects of CoPe projects to collaborative or consultative levels of co-production (Meadow et al., 2015; Chambers et al., 2021; Fleming et al., 2023).

Convergent, co-produced research also requires careful sequencing of research. For example, a computational modeling team cannot advance without specific data inputs from observational environmental scientists, who in turn may rely on community-generated knowledge, which requires time to gather. These interdependencies form a delicate chain in which delays in one area can set off a cascade of delays throughout an entire project, particularly when communication about shifting timelines or expectations breaks down among researchers and community partners. For example, Woodall et al. (2021) found that legal agreements surrounding sharing and curation of data required more time than anticipated.

While some researchers may be anxiously waiting for direction or inputs from community partners, the work of community-engaged research to co-produce new knowledge cannot be rushed because community-engaged research requires repeated and consistent interactions with community partners, time to acknowledge historical harms, space for addressing power imbalances and flexibility for cultivating mutual understanding (London et al., 2020; Luger et al., 2020). What might look like delays to those not intimately involved in the community-engaged aspects of research may instead be taking the time required to carefully translate concepts across knowledge systems and thoughtfully adapt tools to cultural contexts.

Similarly, community members and practitioners can be equally frustrated by the pace of academic research, particularly if there are lengthy delays in sharing findings, applying knowledge to generate solutions or holding community engagement activities to collect data that is meaningful to community partners. Moreover, researchers can be hesitant to release initial findings that have not undergone the peer review process, which then adds additional time – and frustration – for community members and practitioners who are engaged in the research process and awaiting project outcomes.

In addition to the typical temporal challenges of convergent, co-produced research, there is always the possibility that unusual circumstances will further interfere. For example, disruptions can arise from natural disasters, such as hurricanes, or from global emergencies, such as the coronavirus pandemic, which began in 2019. The first round of CoPe awards was made by NSF at the start of the pandemic, which, of course, created unprecedented global challenges. The pandemic also revealed the critical importance of collaborative, adaptive science, particularly in coastal communities where interconnected social, ecological, physical and economic systems amplify vulnerabilities.

### Institutional challenges

Academics conducting co-produced convergence research face multiple institutional challenges as well. For example, academic reward systems celebrate rapid, specialized outputs, while community-engaged work demands long-term investment in relationships and a variety of research approaches. This fundamental mismatch can force researchers into extractive practices or limit them to consultative research, rather than sustainable partnerships yielding co-produced outcomes, perpetuating cycles of distrust and missed opportunities. The very architecture of academic incentives can work counter to engaging in research with the goal of co-producing knowledge (Paynter, 2014). Promotion and tenure committees prioritize peer-reviewed articles, while communities often prioritize actionable white papers or other products. Grant timelines and reporting schedules can clash with the organic timelines of trust-building. These tensions are most acute for early-career researchers, for whom career advancement depends on producing timely, quantifiable results in an institutional context that does not fully recognize the time investment involved in building trusted relationships with community partners and researchers across disciplines and institutions (Foster, 2010; Valentine et al., 2026).

Specific examples of institutional challenges that can hinder co-producing knowledge include the compensation systems designed for traditional research participants, such as stipends that require bank account details or third-party vendor registration, which can exclude those wary of unfamiliar technologies or without bank accounts and the required identifying information. Most of the CoPe-funded projects include multiple universities working on the same CoPe project; when these universities apply different internal rules for participant support and human-subjects research compensation, these bureaucratic inconsistencies can create tangible inequities for community members and practitioners participating in the co-production of knowledge. For example, some CoPe projects experienced differing university budgeting constraints, particularly managing funds across several institutions, which contributed to tensions between research team members and created confusion with community partners about their compensation and roles in the project.

Furthermore, effective co-production of knowledge demands that institutional frameworks enable and support meaningful long-term collaboration. Oftentimes, proposal guidelines underestimate true costs of co-production, including multiple trips to rural areas, stipends for community expertise or translation services. Most importantly, a lack of funding for dedicated engagement staff (community liaisons, project managers, boundary spanners) can leave researchers to carry out these aspects of a project without formal training in cultural brokering and while simultaneously carrying out scientific work. The lack of funding for dedicated engagement team members could also mean that trained engagement staff bounce from funded project to funded project and are unable to maintain relationships that are no longer “funded.”

Additional hurdles may come as teams navigate implementation of IRB requirements. Although these are essential and needed, IRB processes can feel intimidating to community-based organizations unfamiliar with academic protocols and members of research teams who have not typically been involved in human-subjects-based research. Including community-based organizations as research partners on an IRB is critical for the recruitment and data management of human subjects, particularly for underrepresented and marginalized communities; however, the formal nature of these processes, as well as the CITI trainings required, may inadvertently deter potential partners despite their appreciation for the protections IRB provides.

Further, when academic institutions leave no room for the slow work of relationship building and reconciliation, trust gaps can persist or widen. For example, in some cases IRB protocols and other data management requirements (e.g., Tribal data management plans) can take several months to be approved and include unique policies regarding data ownership. These processes, while providing some protections to human subjects and communities, can also pose a challenge when early-career researchers or those mentoring graduate students do not adequately factor in the time needed to complete them or consider the full extent of the effect data management agreements may have on what data will be available for use in publications. Additionally, IRB human subjects policies primarily emphasize procedures that minimize the risk of identifying human subjects, but do not require researchers to undergo trauma-informed or context-based training, which may be necessary to avoid perpetuating historic trauma and further trust issues in some communities (Maldonado et al., 2013; Whyte, 2018; Smith, 2021).

## Recommendations: Strategies for addressing challenges

Co-producing knowledge with coastal communities and researchers from different disciplines can be an incredibly rewarding experience. It can also be intensely challenging, requiring investments of time and energy that extend far beyond more traditional research. From proposal development to project execution, co-produced convergence research requires building collaborative teams and work environments that facilitate careful, relational work that allows participants to understand each other, communicate effectively across disciplines and between researchers and coastal communities, and contribute meaningfully to achieve shared goals. Here, we discuss strategies to address barriers within the four categories identified above and lay the groundwork for successful co-produced convergence research (see Table 2).

**Table 2.** Challenges, strategies and proposed evaluation metrics for co-produced convergence research Table 2. long description.

Nature of challenge	Strategies to address this challenge	Potential evaluation metrics
Epistemological	Bridge linguistic divides by employing inclusive communication practices such as shared glossaries Include boundary spanners on the project team to broker relationships between academic and practitioner spheres Develop team-building activities to facilitate shared understanding	Definitions of key terms and concepts are agreed upon across all research project members and partners Effective boundary spanners are fully integrated within and supported by the project Team building activities are developed and implemented throughout the project life cycle
Relational	Select the style and depth of co-production intentionally Earn trust by being open, reliable and trustworthy Equalize power dynamics among stakeholders, researchers and communities through multiple forms of participatory inclusion	Co-producing stakeholders, researchers, holders of indigenous knowledge and communities can articulate the type of co-production occurring and their role Co-producing stakeholders, researchers, holders of indigenous knowledge, and communities perceive all involved in co-production activities as capable, reliable, having integrity and benevolent Equitable participatory inclusion is determined by stakeholders, holders of indigenous knowledge, researchers and communities and includes factors such as compensation, co-authorship, staff positions and data ownership for non-researchers on the team
Temporal	Embrace the expanded timeline of convergent, co-produced research Acknowledge realistic timelines with community partners and prioritize translation of results Recognize and plan for delays; be flexible Build and sustain partnerships for the long haul Coordinate and track research participation requests	Products desired by stakeholders, researchers, holders of indigenous knowledge and communities are generated early in project life cycle Faster deliverable mechanisms such as short 1-year pilot projects are built into longer multi-year projects Mechanisms to track project coordination with stakeholders, holders of indigenous knowledge, and communities are built into the project from the beginning and are actively used
Institutional	Understand the time needed to build relationships and trust and the associated longer timelines for producing research outcomes Reward and value implementation of best practices in co-produced convergence research Reward and value research products that serve communities’ needs, beyond traditional research products Revise promotion and tenure guidelines to be consistent with points above	Institutional acknowledgment and recognition (e.g., awards, promotion) for convergent and co-produced research Evidence of stakeholder, researcher, holders of indigenous knowledge and community acknowledgment of the value of convergent and co-produced research (e.g., letters of support, continuing, sustained partnerships beyond initial project lifecycle) Revised promotion and tenure guidelines that include convergence research and co-production of knowledge and understanding as having demonstrated impact and value to the academy and institution

### Strategies for addressing epistemological challenges

#### Bridge linguistic divides and employ inclusive communication practices

Effective collaboration in convergence research begins with recognizing language as both a barrier and bridge (Choi and Richards, 2017; Kelly et al., 2019; Klein et al., 2020; Repko and Szostak, 2020; Strober, 2020; Gilligan, 2021; Love et al., 2021). Research teams that monitor terminology gain early insights into group dynamics, as linguistic patterns reveal deeper epistemological divides that can then be addressed. Effective convergence research requires the creation of shared understanding through strategies like recognizing disciplinary differences, identifying a common language and showing respect for diverse perspectives. While there are different strategies for how to achieve this state (Miller et al., 2008; Boon and Van Baalen, 2019, this can be accomplished through inclusive communication practices such as defining terms, avoiding jargon, leveraging translation technologies and actively listening with the intent to understand (rather than to be understood), which can foster a greater sense of belonging and teamwork (Burke et al., 2016; Cooke et al., 2021; Lejano et al., 2021).

Creating shared glossaries or concept maps early on in a research collaboration can clarify terms and expose epistemological differences that require elucidation. Choi and Richards (2017) specifically highlight that epistemic differences and communication styles should be investigated and negotiated so that common ground can be established for research teams. Drawing from our own experiences, for example, one CoPe team^6^ brought together natural and social scientists. In collaboration with coastal community leaders, the team clarified commonly used terms in the context of their project such as *relocation, displacement, managed retreat, migration, immobility* and *adaptation*, among others. Doing so allowed the team to more effectively share knowledge and understand the nuance of using these terms in specific community contexts (for example, the word “managed retreat” can be problematic for Tribal and Indigenous communities due to its association with forced relocation and past injustices).

Another CoPe team^5^, when convening for a two-day workshop, asked participants to volunteer as “jargon watchers.” The role, which rotated among participants, came with an identifying badge and a chime. Whenever the jargon watcher heard the use of jargon, they would ring their chime, prompting the speaker to define the terminology so that everyone in the room could understand what was meant. This brought awareness to the use of jargon in a fun, team-building way, not only reducing its use but also gently encouraging the development of a shared vocabulary.

Equipping researchers with cross-disciplinary skills and knowledge can also help them better understand their colleagues from different fields (Choi and Richards, 2017; Kelly et al., 2019; Klein et al., 2020; Repko and Szostak, 2020; Gilligan, 2021; Love et al., 2021). To foster better understanding across disciplines and approaches, one CoPe RCN team^5^ developed an online Disciplines 101 Seminar Series (C-CoAST, 2026) that brought together scholars from a broad range of fields repeatedly over a period of two years. Each session included two paired 20-minute presentations in which different scholars shared their unique disciplinary “lenses” and approaches in a way that was accessible across fields, inspiring 15-minute discussions following each presentation. Over time, participation in this program facilitated shared understanding, resolved language differences and generated new research ideas and collaborations.

In another vein, some graduate school programs are now preparing students with skills and competencies that are needed for convergence work. Where this is not the norm, requiring graduate students to be co-mentored by faculty representing the student’s primary field of study and another related field may help. This cross-mentoring approach is required by one CoPe hub^2^ and is an essential part of the hub’s establishment of an Individual Development Plan (IDP) for each student funded by the project.

Beyond graduate student training, incorporating brief training sessions on the philosophical foundations of disciplines can help contextualize methodological approaches. For example, one CoPe project^1^ offers regular training to all its researchers – from the most seasoned to undergraduates – on topics ranging from respectful engagement with Tribes, to risk communication best practices, to career advancement. These trainings bring in outside experts who tailor the training to specific CoPe project needs and allow for deep dialogue among CoPe team members after the training. Additionally, using lessons learned from a Coastal Climate Risk and Resilience graduate certificate program, another CoPe project^2^ hosts an annual graduate student and postdoctoral associate summer session which emphasizes science translation skills, network building, face-to-face interactions with stakeholders and across-discipline activities.

#### Include boundary spanners on the project team

To support the creation of a common ground for discussion and understanding, researchers might consider the intentional inclusion of skilled “brokers” between academic institutions or researchers and communities that they serve. Some academics and practitioners already fit the profile of a *pracademic*, or an individual who understands both academic and practitioner experiences and is respected in both spheres (Kuhn, 2002; Posner, 2009; Elbers, 2025; Pousadela et al., 2025).

Pracademics can serve crucial “bridging roles” between each sphere, such as being a network broker, or creating new connections or simply engaging in research that is meaningful across different groups, such as academics, staff researchers and community members (Posner, 2009). By serving in a network broker role, pracademics can assist with assembling and establishing a diverse team of practitioners and community leaders, including cultural mediators and/or other professionals, to work along with academic researchers. Pracademics serving as boundary spanners can mediate discussions and create dialogue among experts and community members (Goodrich et al., 2020). They can also assist in co-design of language protocols to ensure that technical terms align with local meanings and apply participatory methods such as storytelling to bring unspoken assumptions to the surface. Serving as a boundary spanner is not limited to those in the social sciences, as other disciplines in the life and natural sciences can also play a role in knowledge exchange, creation and brokering (Bednarek et al., 2018).

Creating inclusive environments where diverse perspectives are valued and utilized can foster innovation and build trust within teams engaged in convergence research. Boundary spanners and pracademics can and have played a role in achieving this within CoPe projects. For example, many CoPe projects^1,5,6^ include state Sea Grant specialists as part of their teams to fill the boundary spanner role. Sea Grant extension and education specialists are professionals who have long-trusted relationships with the communities in which they live and work. Leveraging these existing relationships can be vital for community-centered research projects.

Additionally, one CoPe project^1^ has a community request portal where any coastal community member or partner can request CoPe research assistance. Requests that fall within the team’s expertise are matched to postdoctoral researchers who dedicate 50% of their time to fulfilling these requests. Requests have ranged from assistance in understanding hazard risk maps for siting community infrastructure to assistance in facilitating and measuring community collaboration and many more (Lai et al., 2023; Mah et al., 2025; https://cascadiacopeshub.org/ccerc/#research). Work resulting from many of the requests has gone on to influence CoPe project research design, demonstrating the iterative nature of co-production.

Another CoPe project^2^ has a Stakeholder Engagement Team that serves in the boundary spanner role. This team provides climate service-like assistance to address needs that are “now” while at the same time liaising between stakeholders and the research team to uncover future research needs. A CoPe RCN^5^, without resources to fund research directly, developed a directory of coastal researchers and practitioners, including contact information for those who wished to share it, facilitating networking and individual requests for collaboration and assistance.

#### Develop team-building activities that build shared understanding

Tangible strategies for increasing knowledge and appreciation of different disciplinary expertise can go a long way toward bridging disciplines (e.g., Choi and Richards, 2017). For example, teams might hold cross-disciplinary work sessions focused on a cross-cutting research topic such as a specific hazard adaptation strategy and metrics needed to measure the effectiveness of that strategy. For example, one CoPe RCN^5^ held three work sessions with researchers from a diverse range of disciplines to consider how to tackle the issue of septic tank failure through convergence research. Similarly, “Open Houses” can allow researchers to quickly learn about adjacent research outside their primary discipline but within the broader team.^1^ These low-stakes activities can plant the seeds of convergence by exploring intersections and insights of how different disciplinary research is or could inform and benefit from a range of disciplinary research.

Furthermore, research teams can greatly benefit from highlighting and celebrating successful convergence research contributions within a large research project and creating space for researchers to connect on a personal level outside of the research environment. Social hours and “ice-breaking” activities can be beneficial by supporting the development of better interpersonal relationships, which improve interdisciplinary team productivity as observed by Love et al. (2021) for one long-term team. When implemented in one CoPe project,^3^ interpersonal activities allowed researchers to get to know their colleagues, which dispelled stereotypes about personality types and disciplinary backgrounds, leading to drastic differences in how participants approach community-based research. In another CoPe project,^2^ activities were intentionally developed to include interdisciplinary “links” from other across-hub team members so that diverse knowledge and input could be brought to bear across all projects.

### Strategies for overcoming relational challenges

#### Select the style and depth of co-production intentionally

Within interdisciplinary research teams where there may be different models of co-production, clear communication is imperative. There are many ways to co-produce research, so having flexibility in the approach, based on the nature of the project and extent of desired community engagement is important. Intentionally selecting a “layered” co-production approach (Welch-Devine et al., 2025) can minimize community research fatigue by ensuring that the deepest levels of engagement are reserved for the efforts that most require or can most benefit from potentially time-consuming contributions from partners. These different approaches reflect what Welch-Devine et al. (2025) describe as co-production approaches that “balance engaging ‘enough’ for co-production to be meaningful but not so much that community members are exhausted” (Welch-Devine et al., 2025, p. 2).

Developing a group-level understanding of the different models of co-production, and the different levels of community engagement each requires, is an essential first step to intentionally selecting the co-production approach(es) that will be employed. One CoPe project^2^ hosted a seminar with research team members and their stakeholder collaborators to discuss the various understandings of “co-production” and what it meant for the project. The resulting decision was that ultimately a “spectrum” of understanding and implementation of co-production processes was the correct across-hub approach, as opposed to having a single definition of co-production (Gonzales, 2019). Across the different CoPe projects, we observe multiple engagement approaches for deliberate co-production; some emphasize deep engagement with key community groups from the initial research design phase, including defining the research questions asked, methods of collection and recruitment of participants (Blockstein et al., 2024), others reflect action research guided by stakeholder-driven social or environmental justice questions.

CoPe projects may also reflect integrated socio-technological models that draw upon collaborative engagement practices and co-produced knowledge from prior projects (Meselhe et al., 2026). However, across these projects, community engagement to co-produce research seems to occur primarily in the research design phase; the degree to which community members and decision-makers are involved in the data analysis processes may vary, even though critical decisions, for example, model proxies or parameters, may occur during this phase (Kolesar et al., 2024).

#### Tailor co-production approaches to the communities involved

Equitable co-production of knowledge requires specific tailoring of approaches based on who is involved. For example, models for Indigenous partnership (David-Chavez et al., 2024) may look very different from models for partnership with other underrepresented and marginalized communities (Homsy and Warner, 2013; Perry and Atherton, 2017; Blockstein et al., 2024). For example, in one CoPe project,^1^ Indigenous partnership included partnering with emergency management to host hazard preparedness events and classes as a way to learn community perspectives and develop a reciprocal relationship with Tribal emergency management. In contrast, within the same CoPe project,^1^ co-production with Latine community members was through local community-based organizations. However, for both communities, reciprocity and eventual co-production of knowledge was built through researcher attendance and assistance at community events and by providing emergency preparedness training to community members.

Within a different CoPe project,^3^ working with the community not only meant working with existing organizations but also creating an organizational structure to facilitate long-term community building that would sustain the project outcomes. The research team worked to address community needs and provide training that helped to support efforts to build community and neighborhood leaders. This assisted in the establishment of rapport with communities that were not necessarily initially trusting of researchers from the institution while providing support to specific community needs, such as developing a neighborhood association and creating a community garden.

Albert et al. (2023) offer nine guiding principles for co-production that can be customized to specific communities of interest. These principles focus on broad underlying relational concepts of power sharing, acknowledgment of many different forms of knowledge and respect and include principles centered on logistics and common sense such as ensuring adequate resources, work flexibility and avoiding jargon. One example of a framework for ethical co-production of knowledge can be found in an assessment of what authors David-Chavez et al. (2024) refer to as a “values-centered relational science model” for social environmental science research that supports Indigenous rights and preserves autonomy over Western science as well as best practices to carry out such work. Gordon and Him (2025) provide a helpful synthesis with extensive suggestions and a framework for “conducting research in a ‘good way’ with Indigenous communities based on their own perspectives as Indigenous researchers.”

Importantly, the term “communities” can take on many meanings within CoPe projects. There are geographic place-based communities (e.g., coastal cities), specific identity or interest communities that often span many geographic communities (e.g., fisherpeople, LGBTQ+, ethnic groups) and Tribal reservations and Indigenous ancestral territories. These often intersecting and overlapping types of communities need to be recognized by research teams, especially with respect to their different capacities and authority to work with researchers. For example, municipalities and Tribal governments may have a department or point person that has the authority to represent that municipality’s or Tribal government’s interests in co-produced research and can make key decisions regarding research design and data management on behalf of the municipality or Tribal government. In contrast, communities of interest often have limited governance structure and authority to make decisions or speak on behalf of the community at large and thus researchers may limit their engagement and efforts to co-produce research with these groups for lack of clear contacts and leadership. Rather than limiting engagement, some CoPe teams^1^ have employed novel strategies to work with a range of different communities of interest such as developing new data management protocols that are shared by identified leaders of specific interest groups and standing up advisory panels to guide research design and implementation for communities of interest.

#### Equalize power dynamics

Balancing power dynamics between researchers and communities so that scholarly knowledge and lived experience are considered equal, requires partnering with communities to redefine research decision-making authority. This can take the form of participatory budgeting or community review boards to allocate resources for community-centered projects, ensuring that community collaborators are compensated equitably for their efforts, pre-negotiating data ownership, authorship of papers and reports and specifying withdrawal rights to ensure that communities can rescind consent without penalty if they believe trust is breached. Several CoPe projects^1,3,6^ integrated multiple forms of participatory inclusion such as co-authorship, paying for conference attendance and travel and creating paid positions related to research, which were informed by lived experiences and addressed community concerns and interests. Through inclusion and integration of community members into the research experience, these projects aim to provide learning opportunities and build a foundation of reciprocity and trust between researchers and community members to foster successful co-creation of knowledge.

Researchers might also consider partnering with or providing information about wrap-around services to community members to aid in their ability to participate. Partnerships may include educational programs (all ages), medical care or health services, financial assistance, translation services, options for childcare, holding events in locations that are easily accessible and recognizing cultural barriers, challenges and appropriateness. For example, in one CoPe project,^1^ the researchers participated in “community cafes” organized by university extension and other community organizations for Latine coastal community members. These “cafes” provided training and information on a range of health care and community services, while the CoPe session focused on hazard preparedness and training and provided information about how to participate in research activities.

#### Earn trust by being open, reliable and trustworthy

Trust is essential to achieving the meaningful and authentic community engagement required for successful co-production in service of community needs. Components of trustworthiness are described in Erickson and Jones (2026) as “Trustworthiness C.R.I.B” as being “capable” (having the knowledge and skills to co-produce knowledge), “reliable” (delivering on promised products), “integrity” (being honest and sincere in all interactions with community members and partners) and “benevolent” (genuinely caring about community members and partners’ well-being). Thus, trust building is a craft that requires time and experience to hone (Erickson and Jones, 2026).

In our collective experience, continuously showing up for community events and building relationships with community members and partners outside of a research agenda demonstrates care and commitment to the communities beyond the project life cycle. Trust is built not only by delivering on the promised products like the one-pagers and the decision-support tools but also by responding and delivering on requests that happen along the way to the generation of those products, such as making connections to other researchers or resources, writing letters of support, offering assistance like proofreading a grant or assisting in the development of an internal survey. These requests may have no clear connection to research project outcomes, but responding to them whenever possible demonstrates researcher interest and investment in community success.

Fostering trust within convergent research teams, like CoPe projects, is also essential. This requires being aware of the policies and processes underlying current injustices and recognizing the power imbalances that often occur between disciplines and within teams based on career stage, social categories of difference and decision-making with regard to the structure, timing and implementation of a project. In one CoPe project,^2^ the creation of a Student and Postdoctoral Leadership Council (SPLC) provided a voice and leadership opportunities for early-career researchers, with representation at the Hub Executive Council level. Furthermore, each project activity is structured such that leadership is provided by the students and postdoctoral researchers, with faculty serving as mentors and counselors.

### Strategies for addressing temporal challenges

#### Embrace the expanded timeline of convergent, co-produced research

When researchers accept and embrace the expanded timeline of convergent co-produced research, they create knowledge that is more valid and more valuable because it emerges from genuine partnership rather than more extractive approaches. The challenge lies not in eliminating temporal tensions, but in recognizing them as markers of quality, the natural pulse of work that bridges academic and community ways of knowing. What gets lost in swiftness is gained in depth, relevance and ultimately, in impact. Likewise, trust is built, maintained and sustained through relationships that develop and grow over time. Systems must be put in place to ensure engagement efforts end when it is appropriate for stakeholders and the research team, not just when funding ends.

Providing preliminary results and regular updates can help alleviate community partner frustration with long research time horizons and ensure that final results are translated into products that the community wants and needs, such as one-pagers, storymaps and policy briefs. Researchers should be cognizant of the need for these community products, work with community members and practitioners to identify what products are desired and prioritize the time and resources to develop these products during the initial stages of research design. Integrating a trusted boundary-spanning organization into a research team is another way to allow the relationships created during co-produced efforts to continue beyond the funding timeline. Boundary-spanning organizations can support and manage interactions and relationships during the project and can actively tend to the partnerships after the funding for specific projects has terminated. Placing an emphasis on aligning different timelines as much as feasible (Woodall et al., 2021) is also valuable.

#### Recognize and plan for delays; be flexible

Just as allowing for timeline flexibility can yield increased depth of learning and relevance of project outcomes, project team members can benefit from anticipating possible delays and considering alternative pathways for progressing through project tasks and objectives. This may be assisted by careful tracking of progress in a way that is visible to all team members and by creating a culture of close communication, perhaps assisted by team communication tools that allow team members to advise of delays promptly so that adjustments can be made. It is also important to be sensitive to the potential for significant disruptions, and to remain flexible and adaptable if major disruptions occur. Considering how to turn a potential negative impact into an opportunity for positive benefit allowed many CoPe projects to thrive despite the pivots required by the pandemic.

For CoPe teams, the pandemic disrupted traditional research timelines as lockdowns delayed fieldwork, modifications to university life required additional time and caregiving burdens increased time away from work, forcing researchers to reevaluate priorities. At the same time, the pandemic fostered team building and innovation as collaborative community-engaged research teams pivoted to online listening sessions and outreach activities, which in many cases expanded their reach and longevity.

#### Build and sustain partnerships for the long haul

Clarity is another cornerstone of sustainable engagement. In areas where multiple projects may appear similar to community members, co-developed short “one-pagers” can distinguish between intertwined efforts, illustrating complementarity and differences. These briefs, created collaboratively with partners as appropriate, outline each project’s unique goals, methods and anticipated outcomes. By demystifying research activities, they can reduce confusion and foster trust, turning potential fragmentation into coordinated, community-aware scholarship. Partnerships can also be built and maintained through low-stakes/high-reward short-term projects. For example, a few CoPe projects^1,6^ employ competitive internal pilot grants that require community partnerships and engagement. These one-year projects allow for quick proof of concept and products such as maps and short reports based on the findings. Through these pilot projects, the CoPe projects have been able to expand community partnerships and demonstrate trust by providing deliverables quickly.

#### Coordinate and track research participation requests

Building equitable research partnerships requires more than good intentions – it demands intentional strategies to avoid overburdening communities while maximizing collaborative impact. It is critical to prevent redundancy and honor prior community contributions. For example, before launching new initiatives, research teams should thoroughly examine existing work in the region (Clark, 2008; Neal et al., 2016; Koen et al., 2017; Kelly, 2021) to ensure their efforts align with, or build on, rather than duplicate or disrupt, what has been done before. One CoPe project^1^ employs the use of “engagement trackers” to identify what researchers are working with specific communities and community partners. Hub researchers are asked to review this tracker before reaching out to potential partners to learn when, by whom and about what a community partner was last contacted. In another CoPe Hub,^4^ the team worked with a community organizer and non-profit director to develop a community organization to assist in coordinating and centralizing efforts to work with community members while simultaneously building community networks and capacity to engage with researchers (Vance-Chalcraft et al., 2024). Another CoPe project^5^ explicitly built upon outcomes from prior community workshops sponsored by a state agency.

### Strategies for addressing institutional challenges

#### Reward and value best research practices

Success in convergence research with coastal communities cannot be sustained without reforms in academic promotion, tenure and recognition processes. Institutional recognition of researchers who are taking the time needed to conduct their work effectively and justly, even when that means a longer timeline, is essential.

#### Reward and value research products that serve communities

Outputs like gray literature reports, policy briefs or community tools, often overlooked in academic evaluations, are real-world relevant and developed not only to be useful, but to be used – considering these outputs as valuable additions to tenure and promotion dossiers is important (Valentine et al., 2026). Equally important to recognize are the extended community collaborations that emerge from co-produced convergence research, yielding sustained future opportunities. Emergence of long-term collaboration is evidenced by community partners providing support letters for projects or launching follow-on initiatives beyond the life cycle of a single grant. These types of less academically tangible, yet deeply meaningful outcomes reflect the growth of trust, knowledge exchange and shared capacity for co-producing advancements in scientific understanding and usable products. They should be reportable in annual reports and tenure and promotion packages and valued along with traditional metrics.

#### Revise promotion and tenure guidelines

While peer-reviewed publications and adherence to project timelines matter, the true impact of engaged scholarship lies in the strength of partnerships, the applicability of research and the long-term relationships and trust built between researchers and communities. Promotion and tenure processes must evolve to recognize those who invest time in collaborative research. We concur with the recommendations of Doberneck (2016), who suggests that universities identify “outstanding examples of scholarly outreach and engagement in reappointment, promotion, and tenure” (p. 16) and celebrate and share them broadly, and to identify successful faculty scholars focused on community-engaged work who can serve as mentors for pre-tenure faculty. Without reforms, institutions risk losing talented scholars who conduct societally relevant, community-engaged, co-productive convergence research and undermining the very partnerships universities seek to foster.

## Conclusions

There are challenges and opportunities in co-produced, convergent coastal research. In lessons learned from designing and implementing CoPe projects, there are both in-group dynamics of research teams and external dynamics with community members that can serve as either disruptors to or facilitators of collaborative projects. We offer strategies from our experience and the literature that can assist in overcoming the epistemological, relational, temporal and institutional challenges involved in this type of work, as well as evaluation metrics that may be useful in assessing the degree to which strategies are being employed (Table 2). In our collective experience, co-produced convergent research can result in deeply rewarding and enlightening experiences that can reshape how we use science to achieve shared outcomes, while also leading us to new understandings that advance fundamental science in important ways. It can also create a significant and lasting benefit to the communities in which we serve and work alongside. To researchers, including those who may be interested but unsure about participating in co-produced or transdisciplinary team projects, we recommend active reflection to assess whether this type of work is the right fit, both in terms of individual ability and willingness to participate and alignment with individual research goals. We strongly encourage interested researchers to explore and participate in co-produced, convergent research.

Institutions aspiring to foster co-produced convergence research must find ways to improve the organizational culture within which such research is conducted and supported. This includes acknowledging the care, labor and time needed to do convergent community-engaged research and reflecting this in tenure, promotion and recognition processes and policies. It also involves encouraging researchers to conduct research in collaboration with non-academics and rewarding the implementation of best practices, and recognizing the additional time required to implement them.

For funders, our collective CoPe experiences point to the value of providing various entry points and staged, progressive funding opportunities to allow time for new community-engaged collaborations to be developed ahead of competitions for large-scale project proposals. In addition, longer grant cycles would be beneficial for both researchers and communities, allowing more time to develop relationships without strict adherence to short deadlines and permitting researchers more time to engage and reflect deeply on the research conducted. This would also provide greater assurance to community members that their investment of time and energy will be well spent with sustained return. We also recommend requiring grant applicants to develop community engagement plans outlining how researchers will include and incorporate community members and their perspectives into the research process, including identification of existing relationships, relationships that need to be developed further and contingency plans to address unforeseen circumstances.

## Data Availability

All data are included in this manuscript through in-text citations, and there is nothing additional to share.

## Table 1. Long description

This table compares six CoPe projects that vary in NSF award type, funding amount, timeframe, geographical scale, goals, key disciplines, and key partners involved in the projects.

Navigate back to Table 1..

## Table 2. Long description

The first column describes the challenges to doing coproduced convergent research. The second column describes the strategies used to address those challenges. The third column describes potential evaluation metrics used.

Navigate back to Table 2..
