# Supplementary material for: Navigating the practice of co-produced convergence research: Lessons from projects funded by NSF’s Coastlines and People (CoPe) program
Source: Camb Prism Coast Futur. 2026 Aug 11;4:e30. doi: 10.1017/cft.2026.10048 (PMC13748884; doi:10.1017/cft.2026.10048)
Supplement: Peer review history

# Reviewer report: Review: Navigating the practice of co-produced convergence research: Lessons from projects funded by NSF’s Coastlines and People (CoPe) program — R0/PR2

**DOI:** 10.1017/cft.2026.10048.pr2

This article is timely and reflects recent efforts to incorporate diverse actors in research. The focus of the article is on how to navigate the complexities of convergent, co-produced

coastal research, a high-stakes field where knowledge production and use depend on deep community partnerships. This includes researchers such as boundary spanners whose focus is the capacity to work directly with community members. The area of focus for the authors are coastal zones, areas of high stress due to sea level rise, coastal inundation, as well as complex economic and social factors. The paper focuses on co-production of convergence research in a variety of settings through NSF’s CoPe initiative, and covers the challenges associated with this type of research as well as how to overcome these challenges through lessons learned through these projects, as well as the literature. This includes epistemological challenges, such as working with different communities and how language can vary between communities, as well as how to overcome barriers to working directly with communities.

Working in this space, my one comment is, what do the authors mean by coproduction in terms of with whom is co-production occurring? Is it the coproduction of the team of convergent researchers or coproduction with the actors and communities where the research is taking place? If that later, were these communities and actors included from the very beginning of the knowledge ideation? This includes working with long-term partners to co-develop research questions, methods, data collection, etc, or were they simply the subjects of the research? Co-production takes long-term interaction and rapport building, and means that the research started at the very beginning, with all actors included. In addition, there must be equity, as noted in the article, as considerations for community members to serve as equal partners. It’s just a comment, as it’s not quite clear. For example, see Ellam Yua et al. 2022, below.

Overall, the authors hit on many important topics in convergent and co-produced research. The article’s flow is good, with challenges at the beginning and a focus on overcoming them through a variety of recommendations.

One clarification is that both convergent and co-production are explained well in the beginning of the article; however, the two are used in tandem throughout the article as one idea or approach, i.e., convergent co-produced research. Maybe a few sentences early on to pull the two approaches together. Why this approach?

This article will make a significant contribution to the literature, and I highly recommend its publication. It’s a strong article, and what I provided here are simply ideas for improvement to assist the reader.

Yua, E., Raymond-Yakoubian, J., Daniel, R. A., & Behe, C. (2022). A framework for co-production of knowledge in the context of Arctic research. Ecology and society, 27(1). https://doi.org/10.5751/es-12960-270134

# Reviewer report: Review: Navigating the practice of co-produced convergence research: Lessons from projects funded by NSF’s Coastlines and People (CoPe) program — R0/PR3

**DOI:** 10.1017/cft.2026.10048.pr3

The article is well written and captures some of the critical challenges of convergence research as well as creative solutions for moving this area of research forward. The literature supporting some of the overarching areas of challenge is good. However, the basis for the insights and recommendations could be presented differently for greater impact. The current format does not appear well suited to a overview review article type.

Given the reliance on the CoPe as the basis for the themes identified in insights and recommendations, I strongly encourage the authors to consider revising this piece as a case study with a program evaluation or science of team science lens. Providing more depth on the data behind the insights and recommendations (e.g., how many projects were examined, size of team per project, level of funding per project) would only enhance the case the authors are making with respect to the things that proved difficult in the process of carrying out the work. I favor the idea of treating this as a case study in part because the idea of making scientific our evaluation of scientific practice is very important. Additionally, the authors seem to be well positioned to draw on a substantial mix of qualitative and quantitative data to achieve this evaluation.

If the authors feel strongly about the overview review, I would consider adding to the insights with other literature or cases so that the review isn’t focused on a singular example (as in, one, US-based funding program) of convergence research. Again, there is good literature support provided in a number of sections, so I am suggesting building this out further to try to extend the examples of where this kind of work has been evaluated. These changes (in either direction) would elevate the contribution of this article to either a state of convergence science review or to a critical evaluation of a case of convergence science.

# Editor report: Recommendation: Navigating the practice of co-produced convergence research: Lessons from projects funded by NSF’s Coastlines and People (CoPe) program — R0/PR4

**DOI:** 10.1017/cft.2026.10048.pr4

**Affiliations:** MaREI, University College Cork, Ireland

Dear Mona

You will see that we have received 2 rather opposite reviews - however, both clearly feel that this is an important contribution that should be published, and I agree with this conclusion. Having read both reviews I am minded that keeping the manuscript as an overview review is probably the better option, but to do so I agree with the comment that the content (discussion) needs to be broader to provide a more comprehenisve context of how this fits into a wider considerationof evaluation. I hope you will revise the manuscript to address the comment on the meaning of co-production/convergence and place your work into a wider context of science evaluation.

Kind regards, Martin

# Editor report: Decision: Navigating the practice of co-produced convergence research: Lessons from projects funded by NSF’s Coastlines and People (CoPe) program — R0/PR5

**DOI:** 10.1017/cft.2026.10048.pr5

**Affiliations:** Geography, Cambridge University, United Kingdom of Great Britain and Northern Ireland

No accompanying comment.

# Editor report: Recommendation: Navigating the practice of co-produced convergence research: Lessons from projects funded by NSF’s Coastlines and People (CoPe) program — R1/PR7

**DOI:** 10.1017/cft.2026.10048.pr7

**Affiliations:** MaREI, University College Cork, Ireland

Dear Authors

Thank you for addressing comprehensively and postively all the comments by the reviewers, which I consider has improved the manuscript and I am happy to recommend that it is accepted for publication.

# Editor report: Decision: Navigating the practice of co-produced convergence research: Lessons from projects funded by NSF’s Coastlines and People (CoPe) program — R1/PR8

**DOI:** 10.1017/cft.2026.10048.pr8

**Affiliations:** Geography, Cambridge University, United Kingdom of Great Britain and Northern Ireland

No accompanying comment.
